# Achieving Self-Directed Integrated Cancer Aftercare (ASICA) in melanoma: protocol for a randomised patient-focused pilot trial of delivering the ASICA intervention as a means to earlier detection of recurrent and second primary melanoma

**DOI:** 10.1186/s13063-019-3453-x

**Published:** 2019-06-03

**Authors:** P. Murchie, J. Masthoff, F. M. Walter, K. Rahman, J. L. Allan, N. Burrows, C. Proby, A. J. Lee, M. Johnston, A. Durrani, I. Depasquale, B. Brant, A. Neilson, F. Meredith, S. Treweek, S. Hall, A. McDonald

**Affiliations:** 1 Academic Primary Care Research Group, University of Aberdeen, Polwarth Building, Foresterhill, Aberdeen, AB25 2ZD UK; 2 Department of Computing Science, University of Aberdeen, Meston Building, King’s College, Aberdeeen, AB24 3UE UK; 3 The Primary Care Unit, Department of Public Health and Primary Care, University of Cambridge, Cambridge, CB1 8SR UK; 4 Aberdeen Royal Infirmary, NHS Grampian, Foresterhill, Aberdeen, AB25 2ZN UK; 5 Health Psychology Group, University of Aberdeen, Health Sciences Building, Foresterhill, Aberdeen, AB25 2ZD UK; 6 Cambridge University Hospitals NHS Foundation Trust, Hills Road, Cambridge, CB2 0QQ UK; 7 University of Dundee, Division of Cancer Research, James Arrott Drive, Ninewells Hospital and Medical School, Dundee, DD1 9SY UK; 8 Medical Statistics Group, University of Aberdeen, Polwarth Building, Foresterhill, Aberdeen, AB25 2ZD UK; 9 NHS Grampian, Dr Gray’s Hospital, Elgin, IV30 1SN UK; 10 Health Services Research Unit, University of Aberdeen, Health Sciences Building, Foresterhill, Aberdeen, AB25 2ZD UK

**Keywords:** Primary care, Melanoma, Cancer, Randomised controlled trial, Survivorship, Self-directed care, e-health, ASICA

## Abstract

**Background:**

Melanoma is common; 15,906 people in the UK were diagnosed with melanoma in 2015 and incidence has increased fivefold in 30 years. Melanoma affects old and young people, with poor prognosis once metastatic. UK guidelines recommend people treated for cutaneous melanoma receive extended outpatient, hospital follow up to detect recurrence or new primaries. Such follow up of the growing population of melanoma survivors is burdensome for both individuals and health services. Follow up is important since approximately 20% of patients with early-stage melanoma experience a recurrence and 4–8% develop a new primary; the risk of either is highest in the first 5 years. Achieving Self-directed Integrated Cancer Aftercare (ASICA) is a digital intervention to increase total-skin-self-examination (TSSE) by people treated for melanoma, with usual follow up.

**Methods:**

We aim to recruit 240 adults with a previous first-stage 0-2C primary cutaneous melanoma, from secondary care in North-East Scotland and the East of England. Participants will be randomised to receive the ASICA intervention (a tablet-based digital intervention to prompt and support TSSE) or control group (treatment as usual). Patient-reported and clinical data will be collected at baseline, including the modified Melanoma Worry Scale (MWS), the Hospital Anxiety and Depression Scale (HADs), the EuroQoL 5-dimension 5-level questionnaire (EQ-5D-5 L), and questions about TSSE practice, intentions, self-efficacy and planning. Participants will be followed up by postal questionnaire at 3, 6 and 12 months following randomization, along with a 12-month review of clinical data. The primary timepoint for outcome analyses will be12 months after randomisation.

**Discussion:**

If the ASICA intervention improves the practice of TSSE in those affected by melanoma, this may lead to improved psychological well-being and earlier detection of recurrent and new primary melanoma. This could impact both patients and National Health Service (NHS) resources. This study will determine if a full-scale randomised controlled trial can be undertaken in the UK NHS to provide the high-quality evidence needed to determine the effectiveness of the intervention. ASICA is a pilot study evaluating the effectiveness of the practice of digitally supported TSSE in those affected by melanoma.

**Trial registration:**

Clinical Trials.gov, NCT03328247. Registered on 1 November 2017.

**Electronic supplementary material:**

The online version of this article (10.1186/s13063-019-3453-x) contains supplementary material, which is available to authorized users.

## Background

Melanoma is common; 15,906 people in the UK were diagnosed with melanoma in 2015 and incidence has increased fivefold in 30 years [1]. Melanoma disproportionately affects younger people with poor prognosis once metastatic [2]. UK guidelines recommend people treated for cutaneous melanoma receive extended hospital follow up to detect recurrence or new primaries [3–5]. However, delivering melanoma follow up to the growing population of melanoma survivors is burdensome for both individuals and health services [6]. Follow up is important, nonetheless, since approximately 20% of patients with early-stage melanoma experience a recurrence and 4–8% develop a new primary, the risk of either is highest in the first 5 years [7–10]. Melanoma can recur locally, regionally or with distant metastases, and new primaries can occur anywhere [11].

It is important to detect new primary and recurrent melanoma as soon as possible. Successful treatment of recurrent melanoma with targeted and immunological treatments is leading to significant improvements in survival even in advanced melanoma [12]. Most recurrences and new primaries are detected by patients between scheduled follow-up visits [3–5]. Thus, guidelines recommend patients conduct monthly total skin self-examination (TSSE) during follow up. An American randomised trial showed that increasing the practice of TSSE in the short-term results in significantly more skin surgery (i.e. in greater detection and tackling of potential melanoma) in people with increased melanoma risk [13].

However, currently people treated for melanoma are possibly missing opportunities for early detection of new primary and recurrent melanoma by not practising regular TSSE or not conducting it effectively. A Scottish study suggests that people delay raising concerns about early recurrence until their next hospital follow-up appointment [14]. There is also evidence from the UK and elsewhere that the practice of TSSE is suboptimal and not practised monthly as recommended by UK guidelines [3–5, 15, 16]. Barriers to initiating and maintaining TSSE include lack of initial training, declining motivation and insufficient time [17]. All of these barriers are tackled by the Achieving Self-directed Integrated Cancer Aftercare (ASICA) intervention.

The ASICA digital intervention supports high-quality TSSE by people with cutaneous melanoma, and appropriate clinical responses when they raise a concern. It is rigorously developed, digitally supported and theoretically based, using specified behaviour-change techniques to prompt users to perform regular TSSE. As reported in more detail elsewhere [18], the intervention was designed to incorporate specific “behaviour change techniques” or BCTs (i.e. the active ingredients that make up an intervention and are required to change behaviour). The BCTs included aimed to develop users’ knowledge and skills about TSSE (e.g. demonstrating the behaviour, rehearsing/practising); enhance/maintain motivation to perform TSSE (e.g. providing information on health consequences of the behaviour, using a credible source for the information); enhance confidence to conduct TSSE successfully (e.g. mastering the skills necessary) and enable self-regulation of action over time (e.g. providing prompts and cues to act, planning when, where, and how to perform TSSE). ASICA may enable earlier treatment and improved outcomes for patients and the National Health Service (NHS) by enabling prompt recognition and treatment of recurrent and new primary melanomas. The ASICA intervention was developed within the Medical Research Council (MRC) complex intervention framework [19] and includes BCTs selected to address constructs of the underpinning theories (information-motivation-behaviour (IMB)) model plus control theory and implementation intentions [20–25]. Development was guided by an expert multi-disciplinary group through several stages [18]. A systematic literature review was conducted, followed by interviews with potential recipients and a facilitated co-design event where all key stakeholders participated in the development of a prototype by acting out a full simulation: a tablet-based digital intervention to prompt and support TSSE, comprising instructional videos and electronic reporting (including photographs) to a clinical nurse specialist in dermatology with subsequent clinical triage [26].

The prototype was further developed in a preliminary 8-month feasibility study to establish its acceptability [18]. Nineteen people treated for melanoma stages 0-2C and who were receiving structured hospital-based follow-up were recruited from six general practices in North-east Scotland [18]. Users were prompted by email each month to undertake TSSE and provide electronic feedback of their findings to the clinical nurse specialist. Qualitative interviews were conducted with the participants after 8 months. Most participants were strongly positive and adhered well to the intervention (*n* = 15/19), and seven participants reported symptoms. Two underwent surgery as a result of participating; one with recurrent melanoma, the second with a benign compound naevus. Intention and confidence to conduct monthly TSSE increased. Issues to improve usability were identified and implemented. We concluded that ASICA is acceptable, safe and effective and offers potential to improve psychological well-being and enable earlier diagnosis of new primary and recurrent melanoma [18]. However, the non-randomised pre-pilot-study gave limited information on recruitment, acceptability, compliance and retention at one site. Also, we collected data on anxiety and depression, but not melanoma worry, and there was wide variability in the scores at baseline and variability in both the magnitude and direction of effect at follow-up, raising the possibility of a bi-directional effect on psychological outcomes that requires further exploration. This study aims to demonstrate that ASICA has the potential to benefit people with melanoma and is feasible to deliver in the NHS.

## Aims and objectives

The aim of the study is to compare a self-directed digital intervention (intervention group) with treatment as usual (control group) in patients treated for a first-stage 0-2C primary cutaneous melanoma within the preceding 60 months.

The hypothesis to be tested is that among patients with melanoma, the ASICA intervention will increase the practice of TSSE in those who use it, compared to controls, without affecting psychological well-being, and will lead to earlier detection of recurrent and new primary melanoma.

The specific study objectives are to:

1. Recruit 240 adult patients with a previous first-stage 0-2C primary cutaneous melanoma.
2. Randomise participants to the ASICA intervention or the control group.
3. Collect baseline patient-reported and clinical data. The baseline participant questionnaire will include the modified Melanoma Worry Scale (MWS) the Hospital Anxiety and Depression Scale (HADs), the EuroQoL 5-dimension 5-level questionnaire (EQ-5D-5 L), and questions about TSSE practice, intentions, self-efficacy and planning.
4. Follow up participants by postal questionnaire at 3, 6 and 12 months after randomisation. Shortened questionnaires (HADS, EuroQoL EQ-5D-5 L, and MWS) will be completed at 3 and 6 months and a full questionnaire, as per baseline, at 12 months.
5. Compare the primary and secondary outcomes between the two arms.
6. Collect data from the intervention group via tablet monitoring, for process evaluation to investigate the frequency and patterning of total skin examination and investigate predictors of sustained skin examination over time.

## Methods

### Study design

**Fig. 1:**
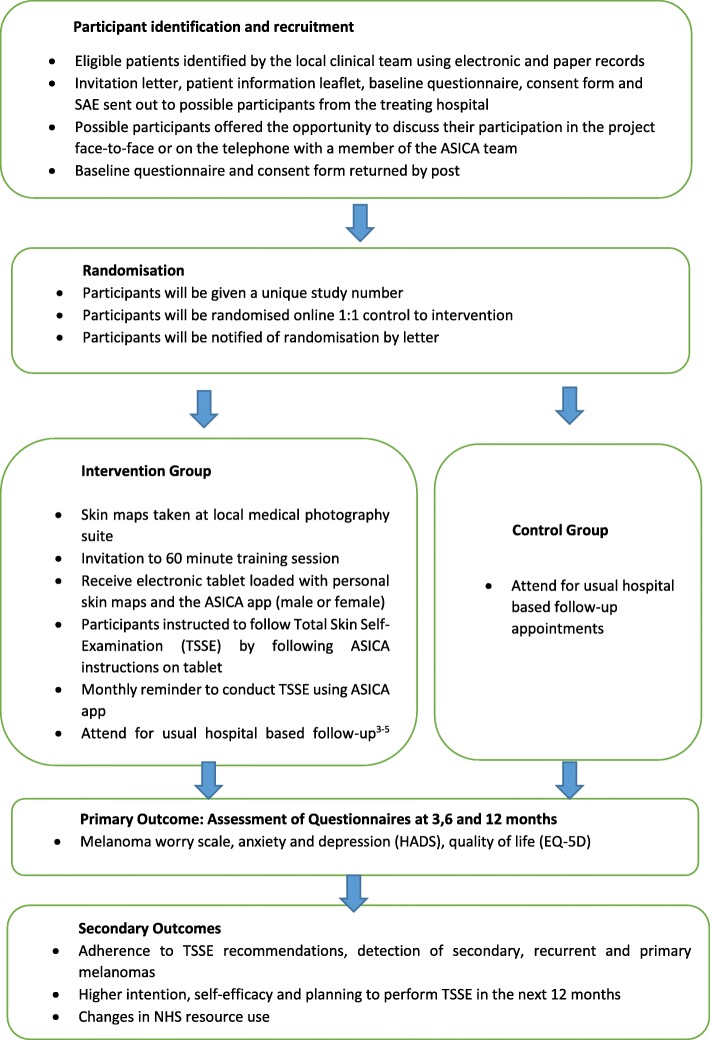
Flow diagram of study design and schedule. SAE, serious adverse event; ASICA, Achieving Self-directed Integrated Cancer Aftercare; HADS, Hospital Anxiety and Depression Scale; EQ-5D, Euroqol 5-dimensions questionnaire; NHS, National Health Service

This is a two-arm, open multi-centre randomised controlled trial (RCT) comparing ASICA, a digital intervention to increase TSSE by people treated for melanoma, with usual follow up. The trial flow diagram is presented in Fig. 1. This protocol follows Standard Protocol Items: Recommendation for Interventional Trials (SPIRIT) guidelines [27]. Participants will be in the trial for 12 months. The primary outcome will be determined up to 12 months after randomisation.

### Study participants

#### Inclusion criteria

We will include adults (age 18 years and over) who have been treated within the preceding 60 months for a previous stage 0-2C primary cutaneous melanoma and can give informed consent.

#### Exclusion criteria

Exclusion criteria are stage 3 and 4 melanoma; previous local recurrence of melanoma within the last 60 months; inability to consent and/or complete questionnaires (e.g. due to cognitive or language issues) and blindness or visual impairment.

### Recruitment

A clinical nurse specialist in dermatology will work with lead clinicians at the two recruiting sites, Aberdeen Royal Infirmary and Addenbrooke’s Hospital, Cambridge. Potential participants will be identified from appropriate sources including, but not limited to, Multidisciplinary Team (MDT) meeting lists, locally held pathology registers and melanoma follow-up clinic registers. Following identification of potential participants, an invitation letter, patient information leaflet (PIL) detailing the trial, consent form, baseline questionnaire and pre-paid return envelope will be sent out directly from the treating hospital. Local contact details will be provided. Potential recruits will be offered the opportunity to meet face to face or have a telephone conversation with a member of the investigative team to discuss their potential participation. Local procedures at the participating hospitals are different and the timing and mode of approaching patients and the consent process may vary in order to accommodate both the specific circumstances at each site and the needs of the patients.

### Informed consent

Potential participants will have the opportunity to discuss all aspects of the proposed research with the local clinical team, family and friends and, if appropriate, with their General Practitioner (GP). Patients who decide to participate will send their completed documents (consent form and baseline questionnaire) in the pre-paid envelope provided to the local clinical team. Participants will be asked to consent to being randomised to receive ASICA or to the control group; to permit the research team to review their secondary-care medical notes at baseline and outcome; to receive the study questionnaires and for future contact to enable longer-term follow up of both groups.

### Randomisation and allocation

Participants will be randomised using a minimisation algorithm based on gender and centre, to minimise imbalance between the intervention and control groups [28]. All participants who enter the trial will be logged with the central study office in Aberdeen and given a unique study number. Participants will be randomised 1:1 control-to-intervention, using the validated remote automated computer randomisation application at the study administrative centre in the Centre for Healthcare Randomised Trials (CHaRT) in the Health Services Research Unit (HSRU), University of Aberdeen. This randomisation application will be available as an Internet-based service. The Principal Investigator (PI), or individual with delegated authority at the site, will access the Web-based system to randomise participants. Participants will be informed of their allocated treatment group after randomisation.

### The intervention that will be evaluated

The intervention will be evaluated as follows:

1. Preparation and training: the intervention group will attend a local medical photography suite to have digital skin images taken. The ASICA intervention app and individual skin-maps will be incorporated within a password-protected individual tablet computer to ensure confidentiality. The app is Android-configured and designed to run on a Samsung Galaxy tablet. The ASICA app comprises (1) an instructional video demonstrating how to conduct a sequential TSSE; (2) a digital skin map of the patient’s own skin; (3) a digital camera and instructions about how to take photographs of skin lesions; (4) a structured electronic TSSE report form, which can be sent to a clinical nurse skin cancer specialist (CNS), which can include photographs of skin lesions that the patient is concerned about. All reports are managed within a secure encrypted server. Participants randomised to the ASICA intervention will be invited to receive their tablet computer at a 60-min group training session held at the local recruiting centre. At these sessions intervention group participants will be instructed how to use the tablet computer and ASICA app to guide themselves through TSSE and send the findings electronically to the study server. Participants will have the opportunity to familiarise themselves with their tablet for a short time before their first skin TSSE. All aspects of using the tablet, from ensuring the internet is connected, detailed step by step instruction, contact details, screen shots of the app to hints and tips on taking photographs with the tablet are contained in a booklet that will be issued to each participant.
2. Prompting to use ASICA: the intervention group will be supported by a clinical nurse specialist in dermatology based in Aberdeen and each month they will be prompted (using the participant’s favoured method of contact (phone, email, text or mail) to conduct a TSSE.
3. Performing and reporting TSSE using ASICA: participants will be asked to follow the TSSE procedure outlined in the integral animated ASICA demonstration and respond using the electronic report form. Where the participant finds a skin lesion that raises concern (either new or associated with their primary site) they will take photographs of the lesion using the camera on their tablet computer. Such photographs will be uploaded and attached to the digital report sent to the clinical nurse specialist.
4. Response by the clinical nurse specialist: the digital reports will be sent to the clinical nurse specialist who will check and log them. The clinical nurse specialist will log all data on TSSE activity (check TSSE conducted, body areas (1–8) checked, concerns noted) and data will be exported into the database for process analysis.
5. Action where the report causes concern: where a patient has registered a concern within their digital report about something they have found during TSSE, the clinical nurse specialist will observe this from the report and contact the patient within 72 working hours for further discussion. They will discuss concerns by telephone in the first instance. Clinical images will be reviewed and further images sent if required. Our pilot study suggests many concerns will be successfully resolved at this early stage.
6. Further clinical action: where discussion and review of clinical images has not fully resolved the concerns of the patient and/or clinical nurse specialist, the clinical nurse specialist will discuss the case and review any clinical images with the site’s lead dermatologist, and an urgent clinic appointment will be arranged.

All participants (intervention and control) will continue to attend their usual structured melanoma follow up as determined by local guidelines throughout the trial. Usual structured melanoma follow up consists of regular (3-monthly or 6-monthly) review appointments with a specialist skin physician or nurse conducted at the secondary care hospital outpatient department. The schedule of appointments is determined by the clinical features of the participant’s primary melanoma.

### Follow-up procedures

Intervention and control group participants will receive postal questionnaires at 3, 6 and 12 months after randomisation. Reminders will be sent to non-responders after 3 weeks. The medical notes of all participants will be reviewed at 12 months and any relevant pathology data will be collected.

### Subsequent arrangements

The local Research Nurse and/or PI will:

1. File a copy of the consent form in the hospital notes along with information about the study.
2. Enter study data on the participant into the bespoke study database hosted by the CHaRT in the Health Services Research Unit, University of Aberdeen.
3. Maintain study documentation at main research office. A copy of the signed consent form will be returned to the Trial Office in Aberdeen.
4. Provide any relevant follow-up clinical data.

Participants will be asked for their preferred mode of contact to be prompted to conduct a monthly TSSE, by phone, post or email. In the case of non-return of questionnaires, attempts will be made by site staff or staff at the Trial Office to trace the participant directly using these means or indirectly by contacting the GP.

### Change of status/withdrawal procedures

Participants will remain in the trial unless they choose to withdraw consent or if they are unable to continue for a clinical reason. All changes in status with the exception of complete withdrawal of consent will mean the participant is still followed up for all trial outcomes wherever possible. All data collected up to the point of complete withdrawal will be retained (with permission) and used in the analysis. The study team will attempt to collect the tablet computer issued to the withdrawing participant.

### Outcome measures

#### Primary outcome

The co-primary outcome measures are the impact of ASICA on worry about melanoma (the Melanoma Worry Scale (MWS)), anxiety and depression (HADS) and quality of life (EQ-5D-5 L) 12 months after randomisation.

#### Secondary outcomes

1. Adherence to TSSE recommendations in the year following the introduction of ASICA.
2. Detection of second primary and recurrent melanoma.
3. Intention and self-efficacy and clearer plans to perform TSSE in the next 12 months. Intention, self-efficacy and plans will be measured using the baseline and outcome questionnaire measures.
4. Pattern of NHS resource use.

### Data collection and processing

**Fig. 2:**
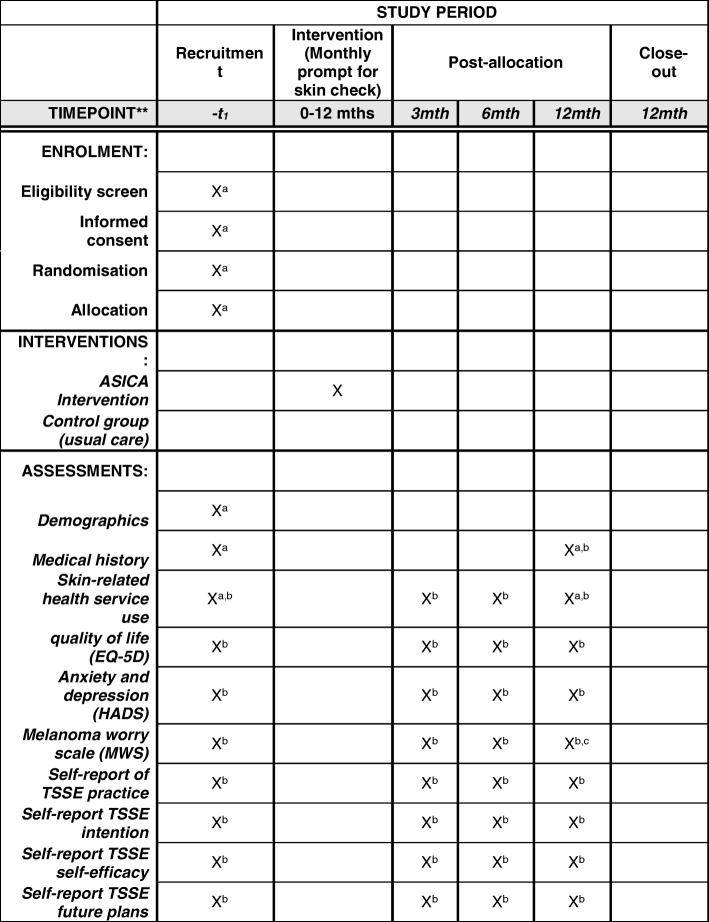
Schedule for assessments/data collection (Standard Protocol Items: Recommendations for Interventional Trials (SPIRIT) [27]. mth, months; ASICA, Achieving Self-directed Integrated Cancer Aftercare; EQ-5D, Euroqol 5-dimensions questionnaire; HADS, Hospital Anxiety and Depression Scale; MWS, Melanoma Worry Scale; TSSE, total skin self-examination

Follow up will continue for 12 months from the date of randomisation. Outcomes will be assessed by participant-completed questionnaires at baseline and 3, 6 and 12 months after randomisation. The research nurse and/or clinician will complete the case report form (CRF) at baseline and at 12 months after randomisation. The components and timing of follow-up measures are shown in Fig. 2 (SPIRIT Figure [27])

### Baseline

A participant visit will not be required to collect baseline data as these will be obtained from participants’ hospital medical records and by postal questionnaire. Participants’ details (age, gender, ethnicity, habitation status, occupation, GP practice, date of melanoma diagnosis, clinical details about melanoma, important comorbidities) will be recorded in the electronic CRF. NHS resource use with respect to skin services will be collected for the 2 years preceding randomisation including number of melanoma follow-up appointments and grade of clinician seen; the number, type and duration of skin-related hospital admissions and the number of additional skin-related hospital outpatient attendances and the grade of physician seen. Participants will complete a questionnaire collecting data on quality of life (EQ-5D-5 L [29]), anxiety and depression (HADS [30]), worry about melanoma (MWS) [31]), self-report of health service use, self-report of TSSE practice (frequency and coverage) and ratings of intention, self-efficacy and planning for future TSSE [32].

### Follow up

Participants will be asked to complete a questionnaire, similar to the baseline questionnaire, to assess outcome measures at 12 months, with a shortened questionnaire at 3 and 6 months after randomisation. These questionnaires will be administered by post or email as preferred by the participant. The research nurse will also re-review participants’ medical records at outcome to collect data on skin-related NHS resource use during the study year, including details of any new skin-related diagnoses and details of melanoma follow-up appointments and other skin-related hospital attendances and admissions. The CRF will enable the clinical nurse specialist to collect relevant baseline and outcome data from secondary care case-notes (including pathology data).

#### Additional data from the intervention group

Adherence to TSSE will be measured using reports of TSSE from the intervention group, which will provide information on the frequency and pattern over time of TSSE, maintenance throughout the 12 months, the time taken and the areas of the body covered. These data will be analysed to assess usage and predictors of usage of ASICA that might be useful in designing improvements to the procedures.

The research nurses will enter locally collected data at the trial centres. Serious adverse events and adverse events will be notified immediately to the PI and recorded by the Trial Office in Aberdeen. Staff in the Trial Office will work closely with the local Research Nurses to ensure the data are as complete and accurate as possible. Study questionnaires will be sent to participants from the Trial Office in Aberdeen and participants will return the completed questionnaires to the Trial Office. Extensive range and consistency checks will be used to further ensure the quality of the data.

## Sample size

A formal power calculation is not needed as this is a pilot trial. However, we have made a pragmatic choice to conduct a relatively large (*n* = 240) pilot study for several reasons. The non-randomised pilot [18] of 19 patients gave limited information on recruitment, acceptability, compliance and retention at one site. We collected HADS (but not MWS) data at baseline and at 6-month follow up. There was wide variation in the scores at baseline and variability in both magnitude and direction of effect at follow up. The feasibility study has therefore raised the possibility of a bi-directional effect on psychological outcomes, with a possible gender interaction, which now needs further exploration in a sample of sufficient size to have representation across the spectra of anxiety and cancer worry scales in both sexes and we believe this will be captured amongst 240 individuals. We need to ensure that ASICA does not adversely affect psychological outcomes and that there will be good adherence to ASICA in most participants, thereby confirming that ASICA could work within the NHS, before proceeding to a definitive trial powered to evaluate harder clinical outcomes (e.g. rates of recurrence and new melanoma, both quantified in the current study). Pragmatically therefore, we believe a sample size of 240 will provide a sufficiently diverse group of participants to provide this information. Although our trial is not powered to detect significant differences in clinical outcomes between groups it will provide valuable information on trial processes and on psychological and clinical outcomes to inform a definitive trial.

## Statistical analysis

The Medical Statistics Team at the University of Aberdeen will be responsible for the statistical aspects of the trial. Data from patient case-notes, on the use of ASICA and from participant questionnaires will be entered into a dedicated secure website. In accordance with Consolidated Standards of Reporting Trials (CONSORT) guidelines, primary analysis will be intention to treat, with a per-protocol sensitivity analysis. No interim analysis is planned. Baseline comparability between the intervention and control group in demographic characteristics, MWS, HADS and EuroQuol EQ-5D-5 L scores, self-reported TSSE practice, intention, self-efficacy and plans and clinical data will be evaluated by examining summary statistics (mean (SD) or median (IQR) for continuous variables, dependent on distribution and number (percentage) for categorical variables). Confidence intervals of between-group differences in the primary and secondary outcomes will be estimated using a repeated measures mixed model, before and after adjustment for potential confounders (including centre, age, gender, deprivation, baseline performance). The confidence intervals generated from the model will subsequently inform a power calculation for a definitive trial. The primary timepoint for outcome analyses will be 12 months after randomisation. The 1-year rate of detection of new primary melanomas, melanomas in-situ, dysplastic melanocytic lesions and recurrent melanomas will be tabulated by randomisation group. Service use and cost data will be summarised by group and compared using a mixed model approach before and after adjustment for pre-specified confounders. The relationship between ASICA usage data, including TSSE trajectories, and outcomes within and between intervention group participants will be examined by multilevel modelling; predictors of usage (including demographic, questionnaire and early TSSE) will be examined in between-participant analyses. Effect sizes of the primary outcomes will be calculated and practical issues affecting the conduct of a definitive RCT summarised. A comprehensive statistical analysis plan will be agreed by the Trial Steering Committee (TSC) prior to any analysis.

## Economic evaluation

A health economist will advise the study statistician on assigning the appropriate unit costs to items of service use for a preliminary assessment of costs. The totality of the data will enable costs, practical issues and effect sizes to be identified for a future definitive RCT of the ASICA intervention. Data collection in the trial will focus on estimating the costs of the intervention and the use of primary or secondary NHS care by study participants. Participant costs will comprise self-purchased healthcare (e.g. prescription and over-the-counter medication related to the skin). Information will be collected using the participant-completed baseline, 3, 6 and 12-month questionnaires. Participants will be asked for information about use of private healthcare. Health service costs incurred as a consequence of the intervention will be recorded. Information on non-protocol visits made to any primary or secondary care provider will be obtained from the questionnaires at baseline and 3, 6 and 12 months and from the secondary care medical record review.

## Process evaluation

In parallel with the quantitative analysis of study data we will conduct a process evaluation of the ASICA intervention and of how it has functioned during the trial. The process evaluation will follow the principles of the Medical Research Council (MRC) process evaluation guidance and will primarily investigate the practical issues around the implementation of the ASICA intervention [33]. It will also consider how the intervention has produced any observed and statistically significant changes between the intervention and control groups with respect to the primary and secondary outcomes. Finally, the process evaluation will consider the impact of context on how the intervention has worked at the two study sites.

## Patient and public involvement

A facilitated co-design event attended by multiple stakeholders and involving five healthy volunteers was conducted in 2015; this helped us to develop the research questions and outcomes for the trial. This was combined with a short feasibility study in which 19 people treated for melanoma used the prototype for 8 months [18]. Patients’ priorities, experience and preferences expressed during these exercises helped to form the research questions and outcome measures for the study. This exercise also helped determine the burden of the intervention for trial participants, with prototype users confirming the feasibility of the intervention. During this trial two patient participants will form part of the Trial Steering Committee; these participants have been consulted throughout the design and recruitment and will continue to meet, receive and discuss reports for the duration of the study. A digest of trial results will be offered to all study participants on completion of the study.

## Research governance, data protection and sponsorship

The trial will be run under the auspices of CHaRT based at HSRU, University of Aberdeen. This will support compliance with research governance, and provide centralised trial administration, database support and economic and statistical analyses. CHaRT is a registered Clinical Trials Unit with particular expertise in running multicentre RCTs of complex and surgical interventions. The Chief Investigator (CI) will, with the support of CHaRT, ensure that adequate systems are in place for monitoring the quality of the trial and appropriate expedited and routine reports, to a level appropriate to the risk assessment of the trial.

Data collected during the course of the research will be kept strictly confidential and accessed only by members of the trial team. Participants’ details will be stored on a secure database under the data protection guidelines and regular checks and monitoring are in place to ensure compliance. Data will be archived to a secure data storage facility. The senior information technology (IT) manager (in collaboration with the CI) will manage access rights to the data set. Participants will be allocated an individual specific trial number and their details will be anonymised on the secure database. We anticipate that anonymised trial data may be shared with other researchers to enable international prospective meta-analyses.

### Dissemination

The findings from the ASICA trial will be disseminated via publication in appropriate scientific journals, presentation at peer-reviewed scientific conferences, and to stakeholders including patients, clinicians, the public and policymakers at appropriate local, national and international meetings.

### Data handling, record keeping and archiving

Essential data will be retained for a period of at least 5 years following close of trial. Electronic data will be archived by CHaRT. The archiving procedures for hard-copy documentation held at local sites will be performed as documented in the Sponsor site agreement. Data will be archived in the Health Sciences Building archive as per Sponsor’s standard operating procures.

### Satellite studies

It is recognised that the value of the trial may be enhanced by smaller ancillary studies of specific aspects of the data, for example differential recruitment from rural and urban areas and predictors of different levels of TSSE adherence. Plans for these will be discussed in advance with the Project Management Group. Sponsor and Research Ethics Committee (REC) approval will be sought for any new proposal, if appropriate.

## Discussion

This study will determine the effectiveness of the ASICA intervention in evaluating the effectiveness of total skin self-examination practice in those affected by melanoma. The study is timely given the growing interest and research activity around digital healthcare interventions in modern health services. Good-quality evidence is needed to inform policy and best practice in the field. This research project will implement and evaluate a rigorously developed and theoretically based digital intervention with real potential to improve patient outcome and the efficiency of services. A relatively large randomised controlled pilot trial is proposed. This will afford the opportunity to establish overall feasibility and trial procedures; however, the sample will be big enough to enable insight into practical issues from the perspective of the different population groups that would take part in a definitive trial and amongst whom the intervention would ultimately be implemented. A further important aspect of digital interventions is the potential impact they could have on wider resource use - in this case there is the possibility of a large increase in the use of skin-related NHS contacts. For this reason, data are being collected to determine any major impact on NHS resources at the earliest possible stage in the evaluation of this intervention. Further, the trial is designed to capture how well potential recipients of a digital intervention can actually use it and to exclude any major psychological impacts in the short-to-medium term. The addition of a structured process evaluation will lend insight into the practical issues of instituting and delivering this intervention. This is especially important given the two proposed study sites. This comes closer to modelling the challenges of implementing digital healthcare intervention than many digital pilot trials and is likely to provide information of value to others conducting research in the field, which goes beyond the specifics of this trial.

### Trial status

Currently recruiting. Participant recruitment began in January 2018 and is expected to finish recruiting in Feb/March 2019. The first participant was randomised on 24 January 2018. Currently approved protocol: Version 2, 1 December 2018.

## Additional file

Additional file 1: Completed SPIRIT checklist for the ASICA trial protocol. (DOCX 24 kb)

## Data Availability

The datasets during and/or analysed during the current study will be available from the corresponding author on reasonable request.

## Abbreviations

AE: Adverse event
ASICA: Achieving Self-directed Integrated Cancer Aftercare
BCT: Behaviour change technique
CHaRT: Centre for Healthcare Randomised Trials
CI: Chief Investigator
CONSORT: Consolidated Standards of Reporting Trials
CRF: Case report form
CTU: Clinical Trial Unit
EQ-5D: EuroQol Group’s 5-dimension health status questionnaire
GCP: Good Clinical Practice
GP: General Practitioner
HADS: Hospital Anxiety and Depression Scale
HRQoL: Health-related quality of life
HSRU: Health Services Research Unit
MWS: Melanoma Worry Scale
NHS: National Health Service
NHSG: National Health Service Grampian
NRES: National Research Ethics Service
PI: Principal Investigator
PIL: Patient information leaflet
PMG: Project Management Group
PQ: Participant questionnaire
R&D: Research and Development
RCT: Randomised controlled trial
REC: Research Ethics Committee
SAE: Serious adverse event
SAP: Statistical analysis plan
SD: Standard deviation
SOP: Standard operating procedure
SPIRIT: Standard Protocol Items: Recommendations for Interventional Trials
TMF: Trial master file
TSC: Trial Steering Committee
TSSE: Total skin self-examination
UKCRC: United Kingdom Clinical Research Collaboration
UoA: University of Aberdeen
